# Bridging the clinical equivalence gap: a nationwide survey on healthcare professionals' perceptions of whole blood-derived versus apheresis platelets in China

**DOI:** 10.1016/j.htct.2026.106453

**Published:** 2026-04-17

**Authors:** Tie Cheng Sun, Ning Zhang, Yu Jie Wen, Di Wu, Wei Dong Zhang, Yan Jun Jia

**Affiliations:** a HLA Laboratory, Beijing Red Cross Blood Center, Beijing 100088, China; b Storage and Issue Section, Beijing Red Cross Blood Center, Beijing 100088, China; c Component Preparation Laboratory, Beijing Red Cross Blood Center, Beijing 100088, China

**Keywords:** Apheresis platelets, Whole blood-derived platelets, Platelet transfusion, Healthcare professionals, China

## Abstract

**Introduction:**

Despite evidence supporting the clinical equivalence of whole blood-derived platelets (WBDP) and apheresis platelets (AP), AP remains the dominant choice for platelet transfusions in many countries. This study aimed to assess Chinese healthcare professionals' preferences, barriers, and conditional acceptance of WBDP as an alternative to AP, especially in the context of platelet shortages and rising clinical demand.

**Methods:**

A nationwide survey targeting physicians, transfusionists, and blood center staff was conducted via the WeChat Mini Program. The survey included 35 questions focusing on attitudes toward AP and WBDP, particularly regarding safety, efficacy, and barriers to WBDP adoption. A total of 781 participants from various healthcare institutions across China completed the survey.

**Results:**

A substantial majority of physicians (78.41%) and transfusionists (93.88%) exhibited a preference for AP compared to WBDP, safety (77.54% and 86.12%) and clinical efficacy (63.77% and 84.9%) as primary factors. Surprisingly, 94.32% of physicians and 89.59% of transfusionists expressed willingness to use WBDP when AP is unavailable, primarily due to blood resource shortages. One of the key findings of this task was that merely 31% of blood center staff and 27% of their hospital customers acknowledged the clinical equivalence between AP and WBDP, with 47% and 53% expressing no opinion. Key barriers to WBDP adoption included concerns over transfusion reactions (92%), efficacy (72%), and safety (68%). To improve the current situation, the following strategies were identified to enhance WBDP adoption: increasing clinical research (60.87%) and physician education (46.96%).

**Conclusion:**

This survey highlights significant reservations among healthcare professionals regarding the clinical equivalence and safety of WBDP. Future efforts should focus on bridging the knowledge gap, optimizing WBDP collection and distribution, and promoting the rational use of both platelet products to address shortages and ensure high-quality patient care.

## Introduction

The demand for platelet transfusions has escalated markedly over the past decade, driven by advancements in medical treatments, an aging population, and the increasing prevalence of conditions requiring platelet support such as cancer therapies and major surgeries [1, 2, 3]. This escalating clinical demand exacerbates supply-chain challenges for healthcare systems worldwide to ensure a stable and sufficient supply of platelet components [4,5]. Platelets, as critical blood products, play a vital role in managing thrombocytopenia and preventing bleeding in high-risk patients. However, the increasing reliance on platelet transfusions has also highlighted the challenges associated with maintaining an adequate supply, particularly in the face of periodic shortages and logistical constraints.

In recent years, there has been a notable shift in many countries toward the preferential use of apheresis platelets (AP) over whole blood-derived platelets (WBDP) [3,4]. Data from the United States National Blood Collection and Utilization Survey Report showed that AP constituted 95.8 % of platelet transfusions in the United States in 2021 [4]. Meanwhile, the proportion of WBDP distributed saw a 28.2 % reduction compared to 2019 [4]. This trend can be attributed to several factors, including the higher remuneration for AP, its perceived superior quality and consistency, and the efficiency of the apheresis collection process. AP enables the collection of higher platelet yields per donor, thereby minimizing dependency on multiple donations and simplifying the processing workflow [6,7]. These advantages have made AP the preferred choice for many healthcare providers, particularly in settings where high-quality platelet products are essential for complex medical conditions [4,8, 9, 10].

Despite the growing dominance of AP, recent studies have demonstrated that WBDP and AP exhibit comparable clinical efficacy and safety profiles [6,10]. Research has shown no significant differences between the two platelet products in terms of bacterial contamination rates, human leukocyte antigen (HLA) alloimmunization, RhD alloimmunization, transfusion-related acute lung injury (TRALI), or febrile non-hemolytic transfusion reactions [6]. The only notable difference is that AP tends to have slightly higher corrected count increments (CCIs), which may be relevant in specific clinical scenarios [10,11]. These findings suggest that WBDP could serve as a viable alternative to AP, particularly in resource-constrained settings or during periods of platelet shortages. However, the widespread adoption of WBDP has been hindered by persistent concerns over its safety, efficacy, and logistical challenges.

In light of these challenges, this study aims to explore the attitudes and perceptions of healthcare professionals in China toward WBDP as a potential alternative to AP. By conducting a nationwide survey, this study seeks to understand the factors influencing the preference for AP, the barriers to WBDP adoption, and the strategies that could enhance its acceptance in clinical practice. The findings of this study will provide valuable insights into the current landscape of platelet transfusion practices in China and inform future efforts to optimize platelet supply chains, mitigate shortages, and ensure high-quality patient care.

## Methods

### Survey design

The present study was designed to evaluate the attitudes and preferences of healthcare professionals in China toward AP and WBDP. A comprehensive survey questionnaire was developed by the authors, comprising 35 questions divided into sections targeting physicians (15 questions), transfusionists (16 questions), and blood center staff (17 questions). The survey aimed to collect data on participants' basic characteristics, preferences for AP and WBDP, concerns regarding clinical application, and the acceptance of WBDP in the absence of AP. To ensure broad representation, the survey was disseminated through the WeChat Mini Program (Questionnaire Star), a prevalent digital survey platform in China, reaching healthcare institutions nationwide, including hospitals, blood centers, and local blood centers. Several questions included the ‘other’ option for free-text responses, which were reviewed by two authors and categorized, with discrepancies resolved by a panel of three additional authors. The survey questions are presented in Supplementary Tables S1–4.

### Survey participation

A total of 781 participants completed the survey, with 86.04 % from hospitals, 10.37 % from blood centers, and 3.59 % from local blood centers, ensuring a diverse representation of healthcare professionals involved in platelet transfusion practices. The survey was conducted from November 11, 2024, to January 20, 2025, and responses were anonymized to protect participant confidentiality. The questionnaire covered key areas such as the participants' basic demographics, their preferences for AP and WBDP, concerns about the clinical use of WBDP, and their willingness to use WBDP when AP is unavailable. This broad participation ensured a comprehensive understanding of the current attitudes and practices related to platelet transfusion across different regions and types of healthcare institutions in China.

### Statistical analysis

Data were analyzed using descriptive statistics (proportions, means ± SD) with Excel 2010 (Microsoft, Seattle, WA, USA) to summarize respondent characteristics, ensuring a robust and systematic approach to data collection and analysis. The survey responses were aggregated and analyzed to identify trends and patterns in healthcare professionals' attitudes toward AP and WBDP. The analysis focused on key factors influencing preferences, barriers to WBDP adoption, and strategies to enhance its acceptance. The use of descriptive statistics allowed for a clear and concise presentation of the findings, providing valuable insights into the current landscape of platelet transfusion practices in China.

## Results

### Geographical distribution and institutional representation of survey responses

**Fig. 1:**
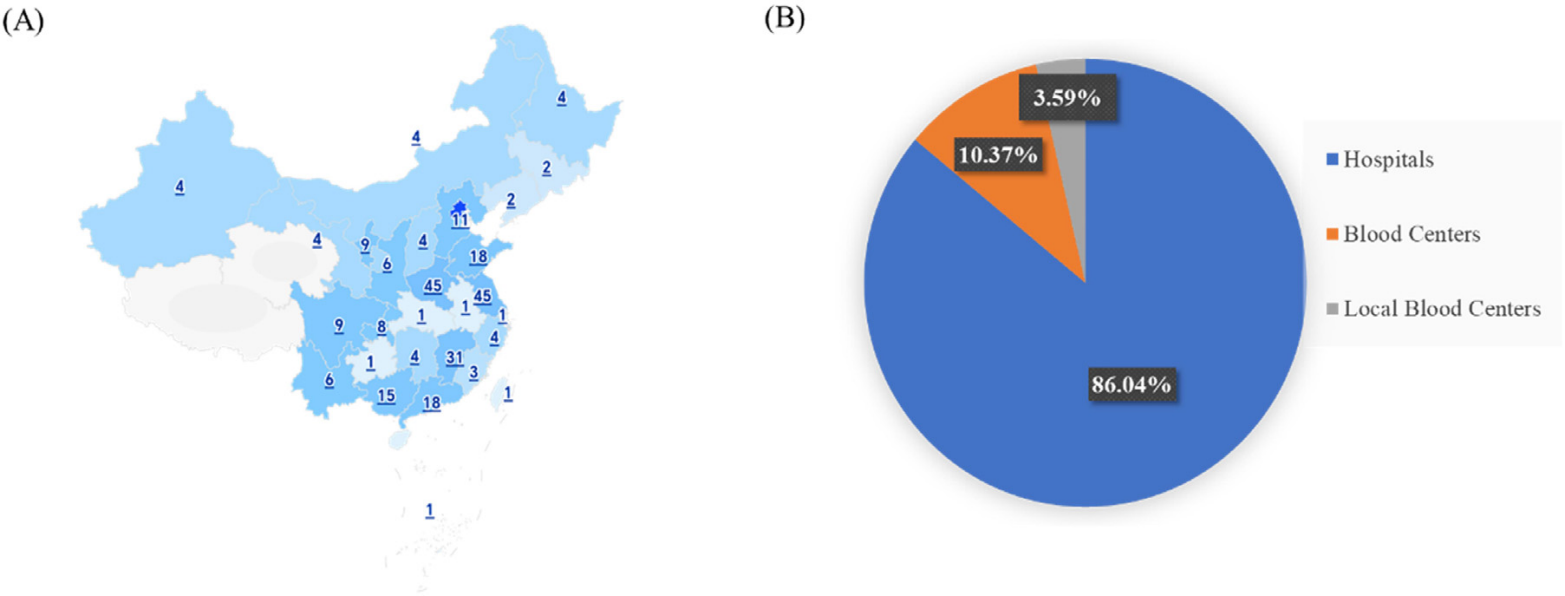
Geographical distribution and institutional representation of survey responses across China: a nationwide analysis of 781 participants. (A) Distribution of participating institutions across different regions of China, illustrating nationwide coverage. (B) Proportion of survey participants categorized by institution type, including hospitals, blood centers, and local blood centers.

A total of 781 participants from various institutions across China completed the survey, ensuring broad regional representation. The geographical distribution of respondents spanned all major regions of the country, as illustrated in Figure 1A. This distribution reflects the central role of hospitals in platelet transfusion practices, while also capturing perspectives from blood collection and distribution facilities. The institutional representation, depicted in Figure 1B, underscores the diversity of respondents, providing a comprehensive overview of attitudes and preferences across different healthcare settings.

### Baseline characteristics of healthcare professionals

The baseline characteristics of the 781 healthcare professionals surveyed are summarized in Table 1. Of the 176 physicians, 65.91 % were female and the mean age was 35.87 ± 9.03 years. The majority held senior (30.68 %) or intermediate (33.52 %) professional titles, reflecting a well-experienced cohort. Transfusionists (*n* = 490) were predominantly female (68.16 %), the mean age was 40.21 ± 9.12 years, and 45.1 % held intermediate professional titles. Blood center staff (*n* = 115) were also mostly female (73.04 %), the mean age was 44.53 ± 9.02 years, and 45.22 % held senior professional titles. These demographics highlight the experienced and diverse nature of the respondents, ensuring that the survey results reflect informed perspectives from professional healthcare in platelet transfusion practices. Here, professional titles follow the typical hierarchy in China’s healthcare system: Junior (e.g., Resident Physician, Technician), Intermediate (e.g., Attending Physician, Supervisor Technician), and Senior (e.g., Associate Chief Physician, Chief Physician, or Senior Technician).

**Table 1:** Baseline characteristics of healthcare professionals across different departments.

Category	n	Level of institution ( %)	Gender ( %)	Age (year) Mean ± SD	Professional title ( %)
Physicians	176	Tertiary (76.71)	Female (65.91) Male (34.09)	35.87 ± 9.03	Senior (30.68)
		Secondary (22.73)			Intermediate (33.52)
		No (0.56)			Junior (35.8)
Transfusionists	490	Tertiary (82.65)	Female (68.16) Male (31.84)	40.21 ± 9.12	Senior (34.9)
		Secondary (16.33)			Intermediate (45.1)
		No (1.02)			Junior (20)
Blood Center Staff	115	Tertiary (48.7)	Female (73.04) Male (26.96)	44.53 ± 9.02	Senior (45.22)
		Secondary (13.04)			Intermediate (36.52)
		No (38.26)			Junior (18.26)

Note: The ‘No’ category indicates that the participant's institution is not classified as a secondary or tertiary hospital (e.g. it is primary healthcare centers or other non-tiered medical facilities).

### Attitudes of physicians toward apheresis platelets and whole blood-derived platelets

**Fig. 2:**
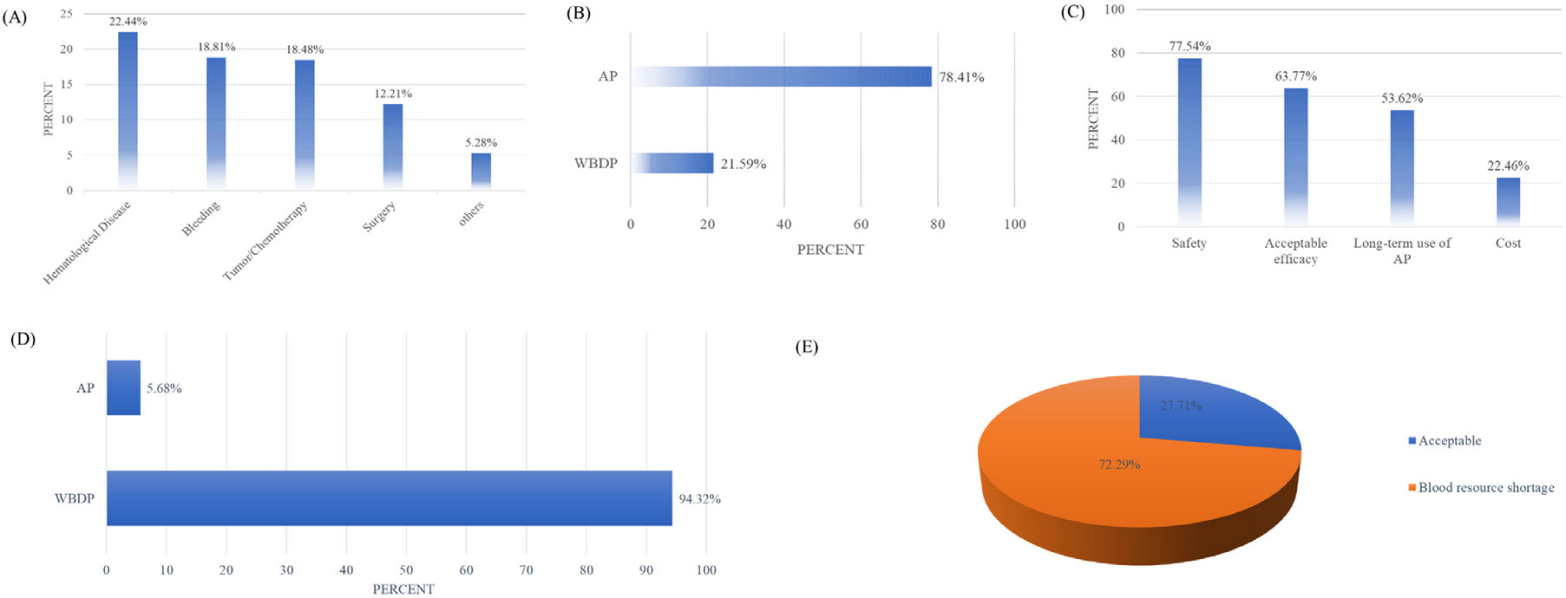
Physicians’ clinical preferences, barriers, and conditional acceptance of whole blood-derived platelets (WBDP): (A) Platelet transfusion indications among patients. (B) Preference for WBDP versus apheresis platelets (AP) in clinical practice. (C) Key factors influencing physicians’ choice between WBDP and AP. (D) Percentage of physicians' willingness (Yes/No) to use WBDP when AP is unavailable. (E) Primary reasons for physicians selecting WBDP as an alternative.

Physicians demonstrated a strong preference for AP over WBDP, with 78.41 % favoring AP for clinical use (Figure 2B). The primary factors influencing this preference were safety (77.54 %) and clinical efficacy (63.77 %), as shown in Figure 2C. These findings underscore the perceived reliability of AP in clinical settings, driven by its established safety profile and consistent efficacy.

When AP was unavailable, 94.32 % of physicians expressed a willingness to use WBDP as an alternative (Figure 2D). However, this acceptance was primarily driven by blood resource shortages, cited by 72.29 % of respondents as the main reason for choosing WBDP (Figure 2E).

### Attitudes of transfusionists toward apheresis platelets and whole blood-derived platelets

**Fig. 3:**
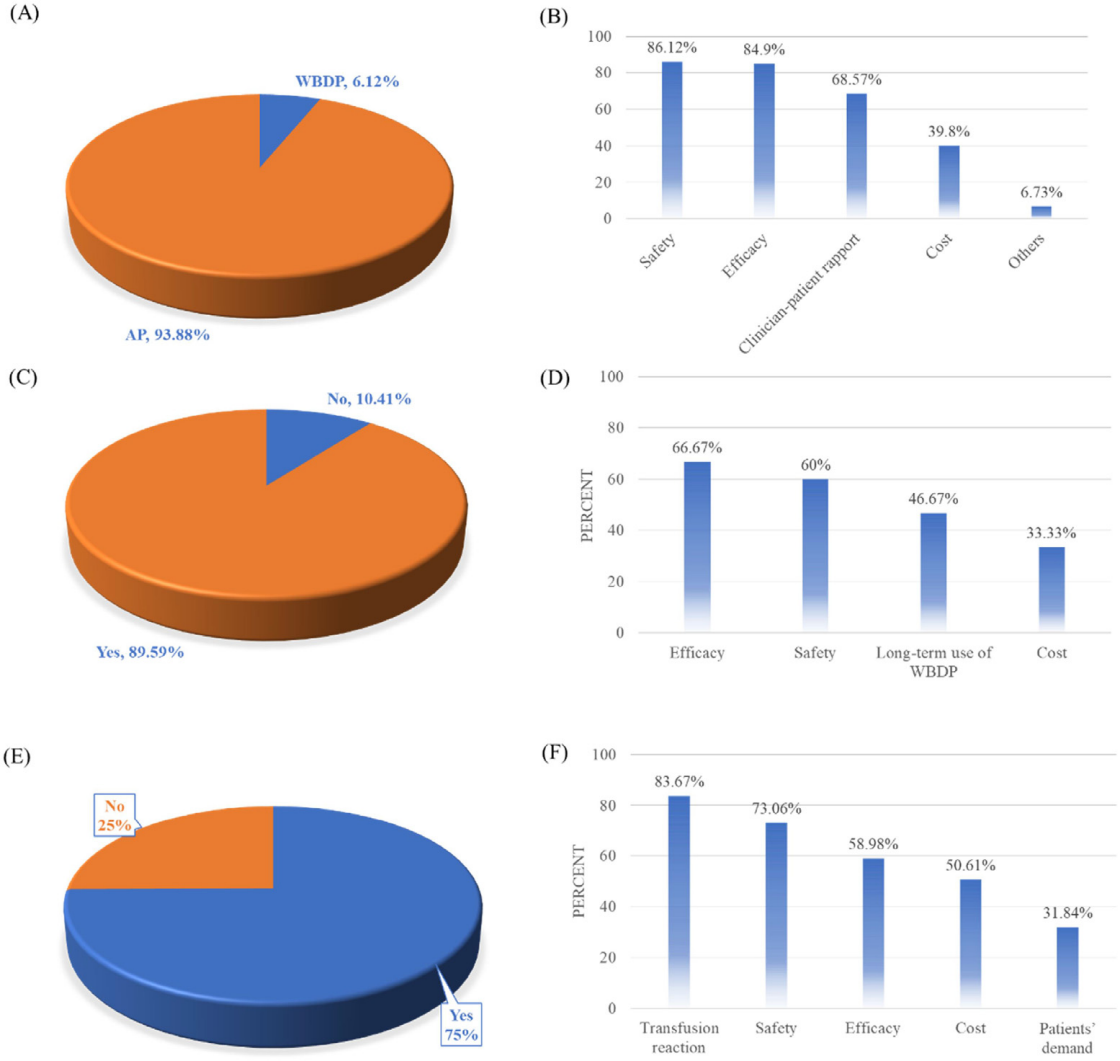
Transfusionists’ platelet source preferences, workload impact, and barriers to whole blood-derived platelets (WBDP) adoption. (A) Distribution of two types of platelet sources chosen by transfusionists. (B) Key factors influencing transfusionists’ decision-making. (C) Transfusionists’ acceptance (Yes/No) of WBDP as an alternative when apheresis platelets (AP) is unavailable. (D) Factors influencing transfusionists' selection of WBDP during shortages. (E) Perceived growth in workload for transfusionists due to WBDP use. (F) Primary barriers to clinical application of WBDP identified by transfusionists.

Similarly, transfusionists exhibited an even stronger preference for AP, with 93.88 % favoring it over WBDP (Figure 3A). Key factors influencing this preference included safety (86.12 %), clinical efficacy (84.9 %), and clinician-patient rapport (68.57 %) (Figure 3B).

Consistent with physicians, a significant proportion of transfusionists would also select WBDP as an alternative only when AP is unavailable in clinical practice (Figure 3C). The primary factors influencing this decision were efficacy (66.67 %) and safety (60 %) (Figure 3D). While this conditional acceptance demonstrates the flexibility of transfusionists in managing platelet shortages, it also highlights the persistent concerns regarding the safety and efficacy of WBDP.

Transfusionists reported a significant increase in workload when using WBDP, with 75 % indicating additional burdens associated with its clinical application (Figure 3E). The primary barriers to WBDP adoption included concerns over transfusion reactions (92 %), safety (73.06 %), and efficacy (58.98 %) (Figure 3F).

### Attitudes of blood center staff to the clinical equivalence of whole blood-derived platelets versus apheresis platelets

**Fig. 4:**
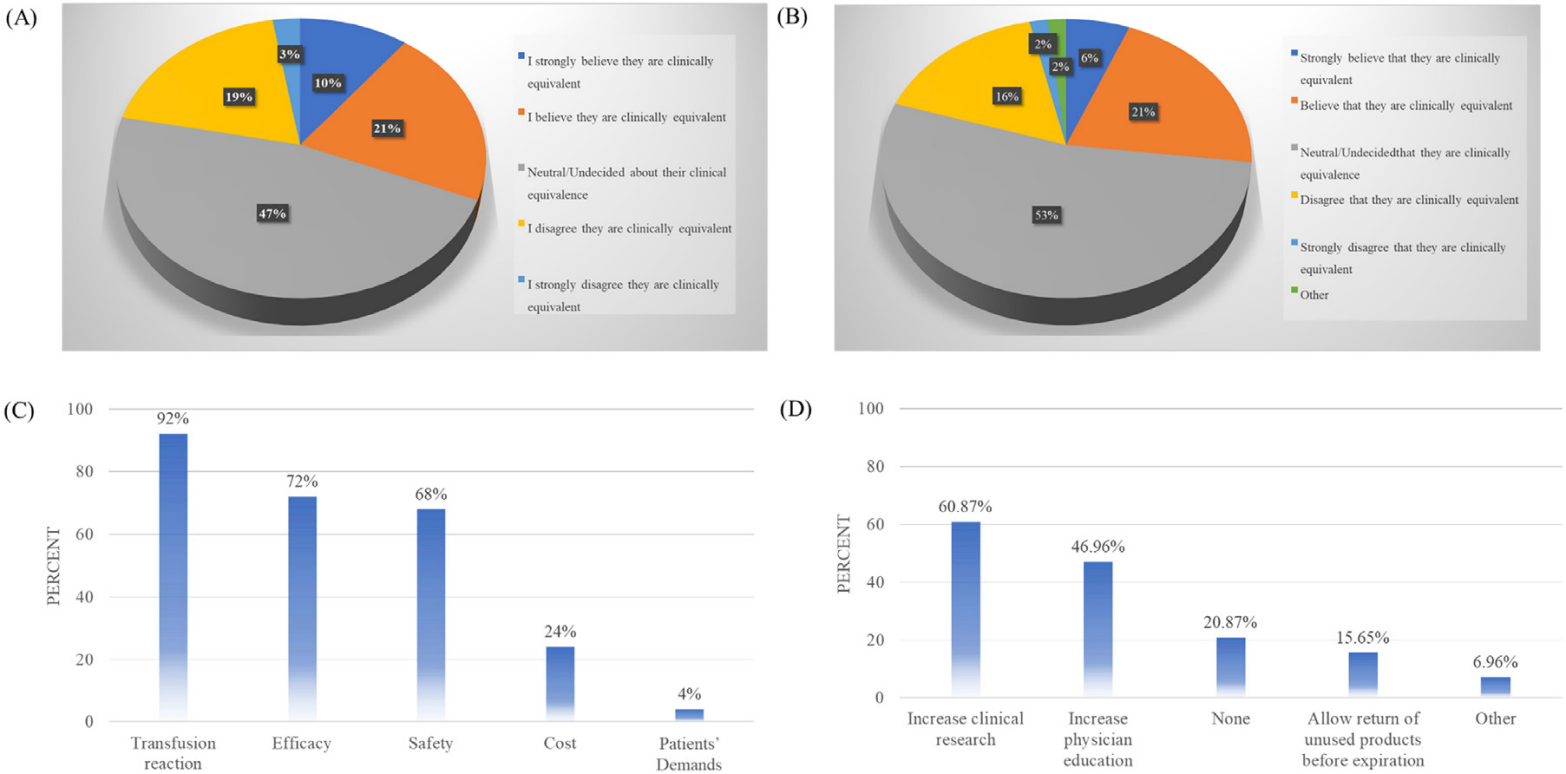
Blood center staff’s perceptions of clinical equivalence and strategies for whole blood-derived platelets (WBDP) adoption. (A) Attitudes toward clinical equivalence of WBDP and apheresis platelets (AP). (B) Perceived hospital customer beliefs on equivalence. (C) Key concerns hindering WBDP adoption. (D) Proposed strategies to enhance WBDP utilization.

The next series of questions was designed to understand the attitudes of the blood center staff toward the clinical equivalence of WBDP in comparison with AP. Of the survey respondents, only 31 % agreed or strongly agreed that AP and WBDP were clinically equivalent. Surprisingly, a significant proportion, 47 %, indicated that they did not have an opinion on their equivalency. Meanwhile, 19 % disagreed and 3 % strongly disagreed that the two products were clinically equivalent (Figure 4A).

Blood center staff were also surveyed regarding their perceptions of hospital customers' beliefs about the clinical equivalence of WBDP and AP. Similarly, only 27 % of the respondents believed that their hospital customers would agree or strongly agree that these products are clinically equivalent, while 53 % thought their customers were either unaware or neutral on the issue. About 18 % felt that their customers did not consider the products to be equivalent (Figure 4B). The remaining respondents generally indicated that they had not specifically asked their hospital customers about their opinions, and some noted that hospitals typically relied on the blood center to decide which product to supply.

The primary barriers to the widespread adoption of WBDP, as perceived by blood center staff, included concerns over transfusion reactions (92 %), efficacy (72 %), and safety (68 %) (Figure 4C). To address these barriers, respondents identified increasing clinical research (60.87 %) and physician education (46.96 %) as key strategies for enhancing WBDP adoption (Figure 4D). Discussion

In China, AP are overwhelmingly preferred over WBDP for clinical transfusions, driven by perceptions of superior safety, efficacy, and long-term clinical experience. The efficiency of AP collection, which generates elevated platelet yields per donation, reinforces its predominance in clinical practice. Conversely, WBDP is often relegated to a secondary option, utilized only during severe blood shortages. Even then, its adoption is constrained by persistent concerns over safety and efficacy, reflecting a persistent skepticism among healthcare professionals. This preference underscores the need to address misconceptions and barriers to WBDP adoption, particularly in light of growing platelet demand and supply challenges.

The data from the present study highlight that the primary limitation to the broader acceptance of WBDP lies in the need to address persistent concerns regarding safety and efficacy. The apheresis process yields higher platelet volumes per donation, reducing donor dependency and streamlining workflows [4,10]. Despite evidence from previous studies demonstrating the clinical equivalence of WBDP and AP in safety and efficacy, this survey reveals that their adoption in China remains conditional, primarily reserved for AP shortages, due to persistent concerns among healthcare professionals. This entrenched preference for AP is sustained by longstanding perceptions of its superiority, reinforced by its standardized collection process. To address this disparity, future initiatives must prioritize evidence-based education to bridge knowledge gaps and optimize the rational use of both platelet products. While AP demonstrates marginally superior clinical CCI outcomes, WBDP remains clinically acceptable, offering cost-effective scalability to address platelet shortages [10,11]. Numerous studies have demonstrated that AP and WBDP exhibit no significant differences in key safety and efficacy metrics, including rates of bacterial contamination, HLA alloimmunization, RhD alloimmunization, TRALI, and febrile non-hemolytic transfusion reactions [6,10]. Despite these robust data, the current survey reveals that a significant knowledge gap persists among physicians, transfusionists, and blood center staff regarding the parity of WBDP with AP. Misconceptions driven by historical preferences for AP, limited dissemination of updated evidence, and fragmented communication between stakeholders hinder the broader adoption of WBDP.

To align practice with evidence, a tripartite collaboration among physicians, transfusionists, and blood center staff is imperative. Firstly, conducting multicenter randomized controlled trials is highly recommended to rigorously assess the clinical equivalence and CCI performance of WBDP. This strategy will provide robust and comprehensive data, ensuring that the outcomes are applicable to a diverse and representative sample of the Chinese population. Leveraging multiple centers will also allow for a broader pool of expertise and resources, thereby enhancing the reliability and credibility of the findings. Secondly, blood centers should optimize centralized WBDP production and distribution to ensure consistent quality and reduce logistical barriers. This requires upgrading infrastructure and adopting standardized protocols for large-scale WBDP preparation. Thirdly, clinical guidelines must explicitly define scenarios for AP versus WBDP use, such as prioritizing WBDP for non-sensitized patients with stable requirements and reserving AP for cases necessitating HLA-matched products or high-frequency transfusions. These guidelines should be codified into national transfusion standards and reinforced through targeted training.

Moreover, educational reforms must address systemic knowledge gaps. Integrating WBDP-focused modules into China’s centralized continuing medical education system will standardize training for physicians, transfusionists and blood center staff. Interactive workshops should emphasize practical decision-making algorithms based on patient profiles, platelet availability, and cost-benefit analyses. Concurrently, blood centers must lead quarterly symposia to update hospital teams on WBDP safety data, production advancements, and inventory management strategies. To sustain progress, institutionalized communication channels are critical. Regional transfusion committees should convene multidisciplinary teams to review WBDP utilization metrics, troubleshoot supply chain inefficiencies, and share best practices. Hospitals must establish real-time feedback mechanisms with blood centers to align production with clinical demand, while transfusionists should lead audits to monitor guideline adherence and outcomes. Encouragingly, the evolving attitudes of blood center staff, as evidenced by their active re-evaluation of WBDP’s clinical role, signal a paradigm shift. By harmonizing evidence-based education, operational collaboration, and policy reform, China can fully leverage WBDP’s potential to achieve platelet supply resilience without compromising patient care.

This study, while providing valuable insights into the perceptions and attitudes of Chinese healthcare professionals regarding platelet transfusion practices, has several limitations that warrant consideration. Firstly, the reliance on self-reported data from participants may introduce response bias, as individuals may not always accurately recall or report their preferences and experiences. Secondly, the demographic and institutional data collected were limited, lacking detailed information such as specific years of experience, exact job roles, hospital size, or blood center capacity, which could influence attitudes toward platelet transfusion practices. Thirdly, the survey was conducted over a specific period, and the findings may not capture changes in attitudes or practices over time or in response to new medical developments or policies. Fourthly, the study primarily used quantitative methods and lacked in-depth qualitative data to explore the reasons behind participants' preferences and concerns. Lastly, the study focuses on healthcare professionals' perceptions and attitudes but does not directly assess patient outcomes related to the use of AP or WBDP. Future research should address these limitations to enhance the robustness and representativeness of the findings, providing a more comprehensive understanding of platelet transfusion practices and their impact on patient care.

## Conclusions

This nationwide survey, the first in China to concurrently analyze the attitudes of physicians, transfusionists, and blood center staff, reveals that despite compelling evidence supporting the clinical equivalence of AP and WBDP, AP remains the overwhelmingly preferred choice. This preference is driven by persistent perceptions of superior safety, efficacy, and long-standing clinical experience, which significantly hinder the broader acceptance of WBDP. To address this gap between evidence and practice, targeted educational initiatives, improved interdisciplinary information sharing, and increased investment in local clinical research are essential to build awareness and trust in WBDP. Furthermore, optimizing the collection, storage, and distribution processes for WBDP is crucial to alleviating platelet shortages without compromising patient care. Future efforts must prioritize closing these knowledge gaps, promoting the rational, context-specific use of both platelet products, and fostering a collaborative, multi-stakeholder approach to build a more resilient and sustainable platelet supply system.

When interpreting these findings, several study limitations should be acknowledged. First, the reliance on self-administered questionnaires may introduce response bias, as reported preferences may not fully align with actual clinical behavior. Second, the collected demographic and institutional data were limited; more detailed covariates could offer deeper insights into the drivers of preference. Most importantly, this study assessed professional perceptions but did not evaluate patient-centered outcomes associated with AP versus WBDP use. Future research should incorporate direct measures of clinical efficacy, safety events, and patient recovery to bridge this critical gap between professional perception and patient reality. By addressing these challenges, healthcare systems can move toward a more evidence-based and balanced platelet transfusion strategy, ultimately aiming to improve patient outcomes and mitigate the impact of supply shortages.

## Data availability

The data that support the findings of this study are available from the corresponding author upon reasonable request.

## Funding

None.

## Conflicts of interest

The authors declare no conflict of interests.

## Appendix Supplementary materials

Image, application 1

## Footnotes

Supplementary material associated with this article can be found in the online version at doi:10.1016/j.htct.2026.106453.
