# Supplementary material for: Bridging the clinical equivalence gap: a nationwide survey on healthcare professionals' perceptions of whole blood-derived versus apheresis platelets in China
Source: Hematol Transfus Cell Ther. 2026 Apr 17;48(3):106453. doi: 10.1016/j.htct.2026.106453 (PMC13094495; doi:10.1016/j.htct.2026.106453)
Supplement: Supplementary file 1 [file mmc1.docx]

**Table S1:** Shared Questions

| **No.** | **Question** | **Answer Options** |
| --- | --- | --- |
| 1 | What type of institution do you work at? | Hospital, Blood Center |
| 2 | What is the level of your medical institution? | Tertiary, Secondary, No |
| 3 | What city do you work in? | Free text |
| 4 | What is your gender? | Male/Female |
| 5 | What is your age? | Free text |
| 6 | What is your professional title? | Junior Title, Intermediate Title, Senior Title |
| 7 | What is your profession? | Physicians, Transfusionist, Blood Center Staff |

Then proceed to the relevant section based on your profession (physicians: Questions 1-7 and 8-15; Transfusionists: Questions 1-7 and 16-24; Blood Center Staff: Questions 1-7 and 25-35)

**Table S2:** Clinicians’ Responses Section

| **No.** | **Question** | **Answer Options** |
| --- | --- | --- |
| 8 | What disease is the patient receiving platelet transfusion for? | Bleeding, Surgery, Hematological Disease, Tumor/Chemotherapy, Others |
| 9 | Which do you prefer to use: WBDP or AP? | WBDP/AP |
| 10 | If choosing WBDP: Reasons? | Long-term use of WBDP, Acceptable efficacy, Safety, Cost |
| 11 | If choosing AP: Reasons? | Long-term use of AP, Acceptable efficacy, Safety, Cost |
| 12 | If only WBDP is available, would you choose to use it? | Yes/No |
| 13 | If yes, why? | Acceptable, Blood resource shortage |
| 14 | If no, why? | Safety, Efficacy, Patients’ demand, Cost |
| 15 | If only AP is available, what would you consider more? | Safety, Cost, Efficacy, Other |

**Table S3:** Blood Transfusion Department Physicians' Responses Section

| **No.** | **Question** | **Answer Options** |
| --- | --- | --- |
| 16 | Which do you prefer to use: WBDP or AP? | WBDP/AP |
| 17 | If choosing WBDP: Reasons? | Long-term use of WBDP, Acceptable efficacy, Safety, Cost |
| 18 | If choosing AP: Reasons? | Long-term use of AP, Acceptable efficacy, Safety, Cost |
| 19 | If only WBDP is available, would you choose to use it? | Yes/No |
| 20 | If yes, why? | Acceptable, Blood resource shortage |
| 21 | If no, why? | Safety, Efficacy, Cost |
| 22 | If only AP is available, what would you consider more? | Safety, Patients’ demand, Cost, Efficacy, Other |
| 23 | Do you think choosing WBDP would increase the workload for cross-matching? | Yes/No |
| 24 | From the perspective of the Blood Transfusion Department, what do you think is the biggest obstacle to the clinical use of WBDP? | Safety, Transfusion reaction, Patients’ demand, Cost |

**Table S4:** Blood Center Staff Responses Section

| **No.** | **Question** | **Answer Options** |
| --- | --- | --- |
| 25 | Does your blood center currently provide Whole Blood Derived Platelets (WBDP)? | Yes/No |
| 26 | How many units of WBDP did your unit produce in the last year? | Free text |
| 27 | If no - Have you ever produced WBDP before? |  |
| 28 | If yes - Why did you stop producing WBDP? | Sufficient staffing, Bacterial reduction, Cost, Efficacy, Logistics/inventory management, Safety, Other - Please specify |
| 29 | If no - Why have you not produced WBDP? | Sufficient staffing, Bacterial reduction, Cost, Efficacy, Logistics/inventory management, Safety, Other - Please specify |
| 30 | As a blood center physician, do you believe that Apheresis Platelets (AP) and Whole Blood Derived Platelets (WBDP) are clinically equivalent and interchangeable? | I strongly believe they are clinically equivalent, I believe they are clinically equivalent, Neutral/Undecided about their clinical equivalence, I disagree they are clinically equivalent, I strongly disagree they are clinically equivalent |
| 31 | If you disagree or strongly disagree - What are your concerns? | Safety, Transfusion reaction, Cost, Patients’ Demands. |
| 32 | How would you describe your hospital's belief that AP and WBDP are clinically equivalent? | I strongly believe they are clinically equivalent, I believe they are clinically equivalent, Neutral/Undecided about their clinical equivalence, I disagree they are clinically equivalent, I strongly disagree they are clinically equivalent, Other - Please specify |
| 33 | If there are any such concerns, what measures can your blood center take to alleviate hospitals' concerns about WBDP? | Allow return of unused products before expiration, increase clinical research, increase physician education, None, Other - Please specify |
| 34 | Given the recent platelet shortage, have you considered or started producing WBDP to address the shortage? | We are already producing WBDP, we have started producing WBDP due to the shortage, we are considering producing WBDP due to the shortage, we are not considering producing WBDP due to the shortage |
| 35 | If we are already producing or have started producing - Would providing WBDP pools help alleviate the shortage? | Yes/No |
